# The unpredictable resorption of bioresorbable scaffolds—A tale of two ABSORBs

**DOI:** 10.1002/ccr3.9010

**Published:** 2024-05-31

**Authors:** Akshyaya Pradhan, Shubhajeet Roy, Monika Bhandari, Pravesh Vishwakarma, Marco Alfonso Perrone, Rishi Sethi, Md. Al Hasibuzzaman

**Affiliations:** ^1^ Department of Cardiology, Lari Cardiology Centre King George's Medical University Lucknow India; ^2^ Gandhi Memorial and Associated Hospitals, King George's Medical University Lucknow India; ^3^ Department of Cardiology and CardioLab University of Rome Tor Vergata Rome Italy; ^4^ Institute of Nutrition and Food Science, University of Dhaka Dhaka Bangladesh; ^5^ The First Affiliated Hospital of Ningbo University Ningbo Zhejiang China

**Keywords:** ABSORB‐GT 1, bioresorbable scaffold, optical coherence tomography, resorption, vasomotion

## Abstract

Bioresorbable stents represent a revolutionary treatment for coronary artery disease. Such a device offers the prospect for complete naturalization of artery lumen after strut resorption and restoration of vasomotion while curtailing the duration of dual anti‐platelet therapy. The prototype bioresorbable scaffold (BRS—ABSORB GT1) demonstrated good feasibility and safety in the initial studies compared to metallic drug eluting stent but later fell out of favor due to multiple report of stent thrombosis and target lesion failure. Unpredictable resorption of struts turned out to be one of the “Achilles heel” of the BRS and stent strut were still visible in vessel on optical coherence tomography (OCT) at 3 years. We report a case of differential resorption of two ABSORB BRS implanted simultaneously in the same patient by the same operator. Follow up coronary angiogram revealed only minimal plaques on right coronary artery (RCA) and left anterior descending artery (LAD). The BRS were identified on cine‐angiogram by their radio‐opaque markers at both ends. The OCT run in LAD artery revealed “ghost remnants” of BRS struts in LAD, whereas the RCA BRS had completely healed with minimal “ghost” struts. The ghost remnants of BRS resembled the original “Check box” appearance on OCT during the index implantation.

## 1 INTRODUCTION

Percutaneous coronary intervention with drug eluting stents (DES) has revolutionized the contemporary management of coronary artery disease. Despite the improvement in early outcomes, long‐term challenges of restenosis and late stent thrombosis remain with metallic DES. Bioresorbable scaffold (BRS) remain a potentially attractive solution to these problems with the dual benefits of curtailment of long‐term risk of restenosis and obviating the need for long‐term dual anti‐platelet therapy (DAPT). However, the only clinically approved BRS—ABSORB GT1 failed to produce clinically meaningful long‐term outcomes after initial encouraging feasibility and safety data from various trials and registries. The product was withdrawn form market in 2018 by Abbott Vascular owing to low sales demand. The basic concept behind the genesis of BRS technology, that is resorption of stent struts was very unpredictable, with various researchers reporting varied time intervals using ABSORB BRS. This could have been affected by variable operator skills, nonuniform procedural aspects, individual patient factors and last but not the least device related factors. We report a case of differential resorption of two ABSORB stents (demonstrated on OCT) implanted on the same day, nearly at the same time and by the same operator in the same patient.

## 2 CASE REPORT

A 57‐years‐old gentleman presented to the emergency unit of the Cardiology Department with mild chest pain. He had a history of acute non‐ST elevation myocardial infarction 5 years back for which he underwent coronary angiogram and two BRS were implanted one each in left anterior descending (LAD) and right coronary artery (RCA). The implant sizes in LAD and RCA were 3.5 mm × 12 mm and 3.5 mm × 18 mm respectively. A third metallic DES was implanted in left circumflex artery as a staged procedure 3 months later. His left ventricular systolic function was mildly reduced (LVEF = 48%), and apex as well as distal septum were hypokinetic. A review of the old records revealed that both the BRSs were implanted on the same day, in the same sitting and interestingly by the same operator. The patient was on DAPT (Aspirin & Clopidogrel each 75 mg) with an additional secondary prevention therapies as directed by the prevalent guidelines. We decided to perform a check angiogram as well as an optical coherence tomography (OCT) evaluation of both vessels. His routine biochemistry was normal. A 6Fr right radial access was used and coronary ostia were engaged with routine workhorse guide catheters. After angiograms were over, a routine guidewire was used to cross the scaffold. OCT runs were carried out using the Dragonfly catheter (St. Jude Medical, Minnesota, MN, USA) with standardized techniques.

**FIGURE 1:**
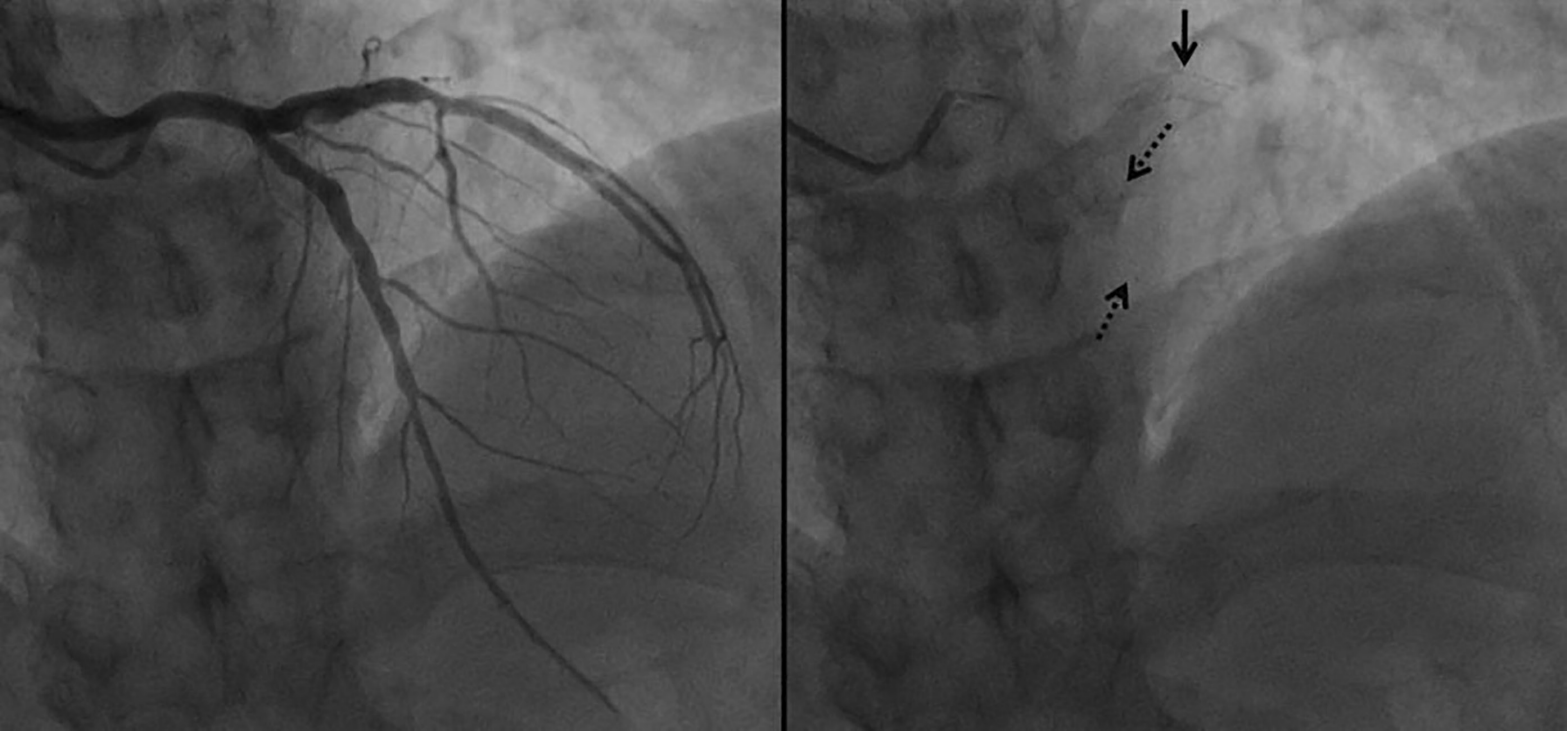
Coronary angiogram of left anterior descending (LAD) artery in anteroposterior projection with cranial angulations showing a patent bioresorbable scaffold (BRS) in LAD(left panel). The right panel depicts the radio‐opaque markers at both ends of BRS (dashed black arrows) by virtue of which it can be recognized on fluoroscopy. The solid black arrow depicts the easily discernible metallic drug eluting stent in left circumflex artery.

**FIGURE 2:**
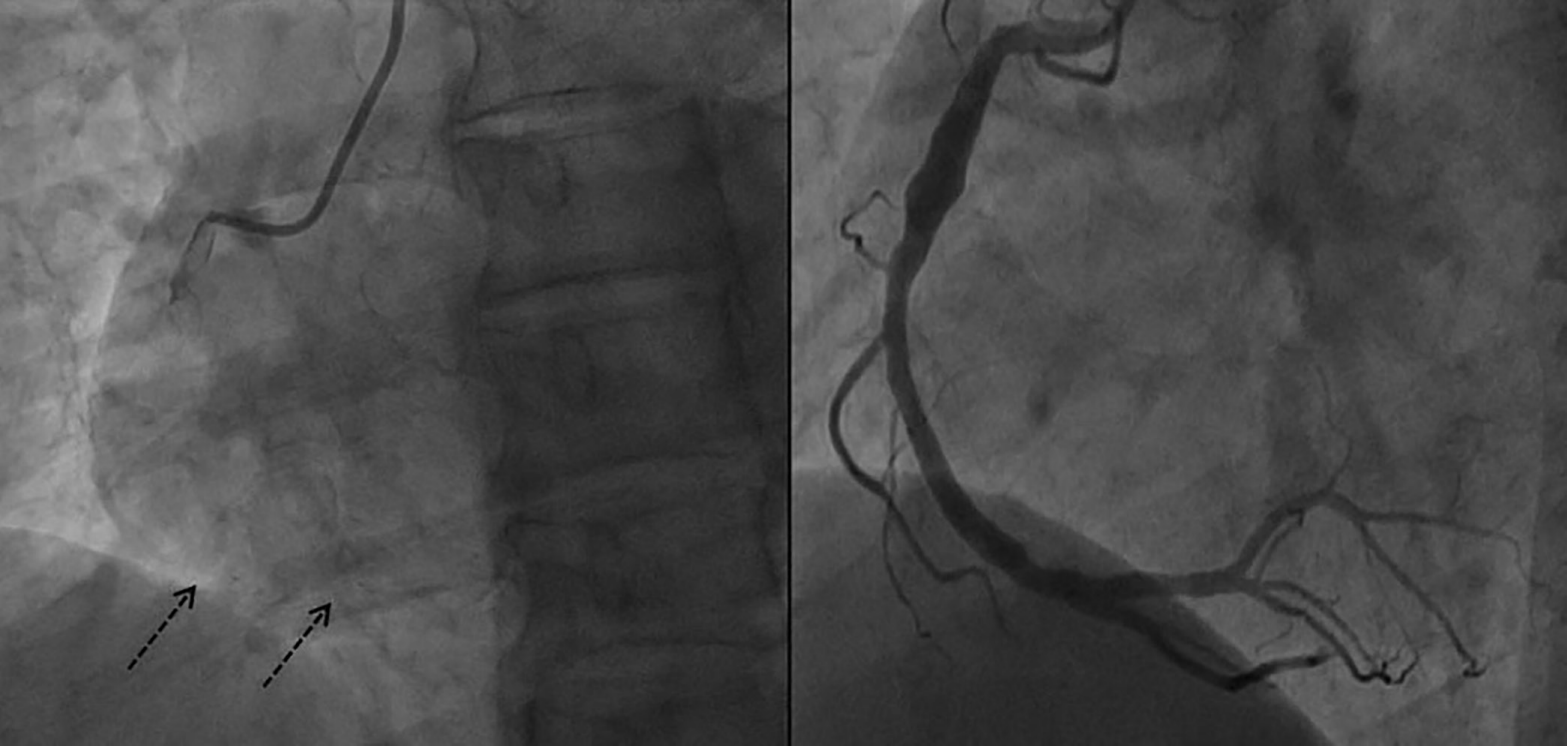
Coronary angiogram of right coronary artery in left anterior‐oblique projection with cranial angulation showing a patent bioresorbable scaffold (BRS) and ectatic segment in proximal part of vessel (Right panel). The left panel depicts the radio‐opaque markers at both end of BRS (black arrows) by virtue of which it can be recognized on fluoroscopy.

**FIGURE 3:**
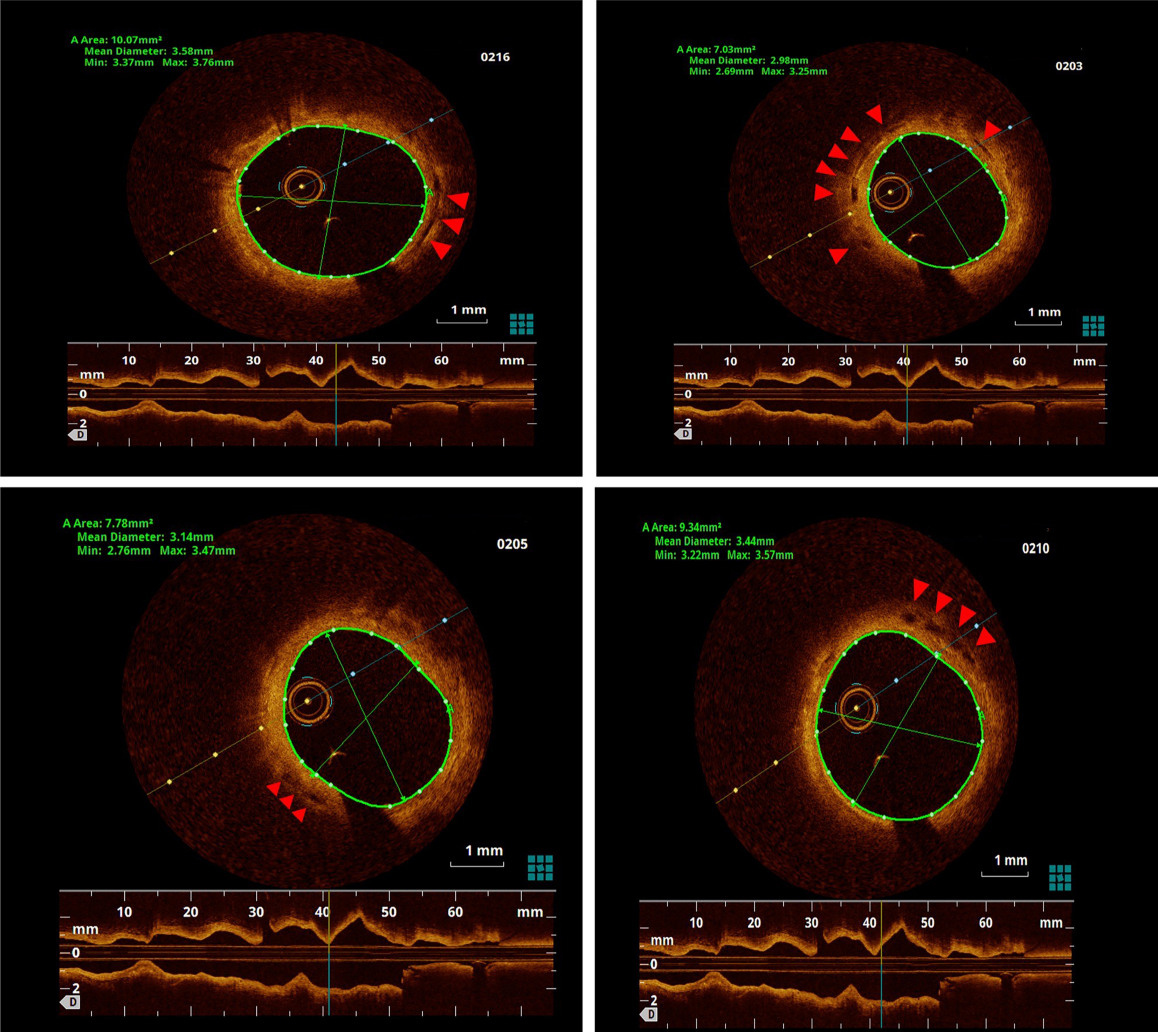
Optical coherence tomography runs at different sections of left anterior descending artery demonstrating the unresorbed struts of bioresorbable scaffold (red markers) seen a lucent area inside the hyper‐refractive endothelium.

**FIGURE 4:**
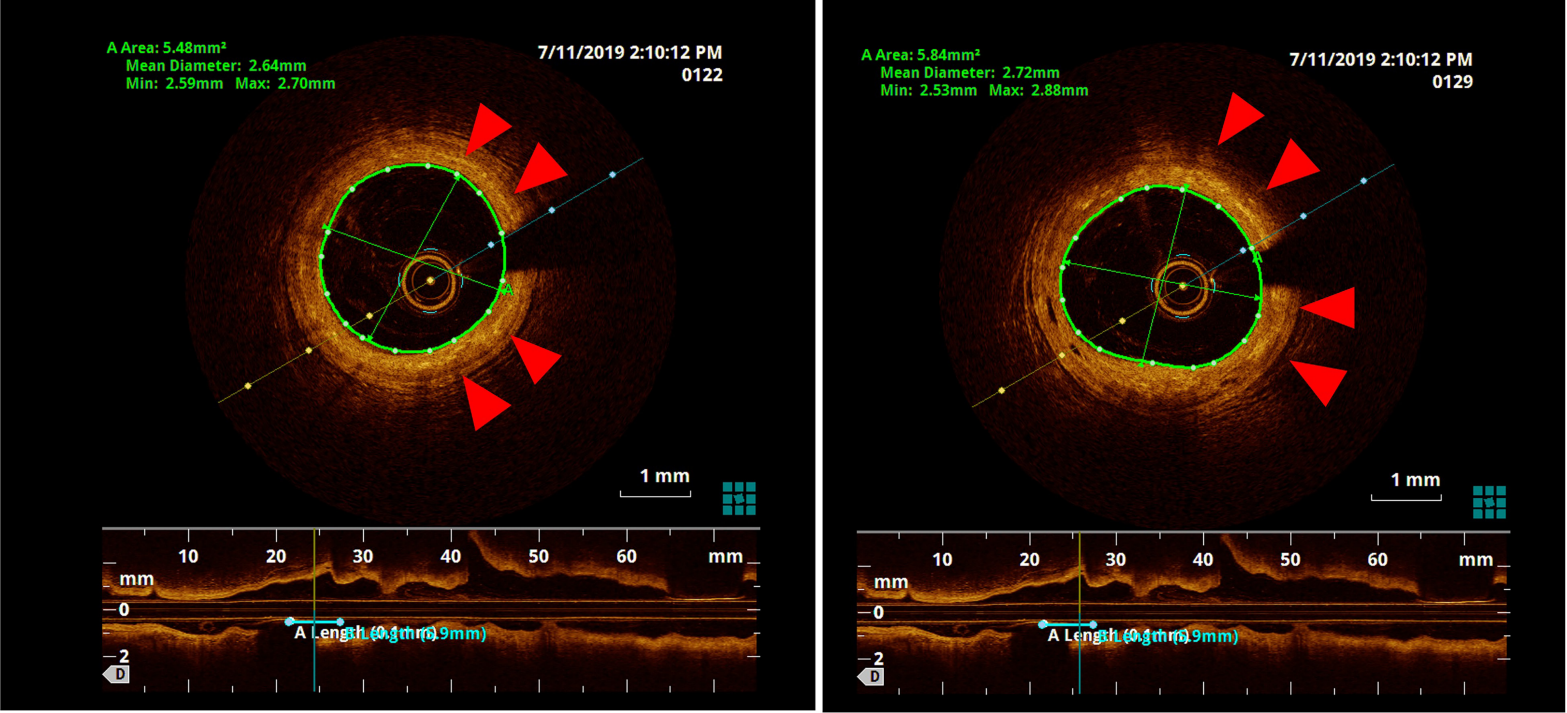
Optical coherence tomography runs at different sections of right coronary artery demonstrating the complete resorption of bioresorbable scaffold (red markers) with only meager lucent areas in the bright/hyper‐refractive endothelium.

**FIGURE 5:**
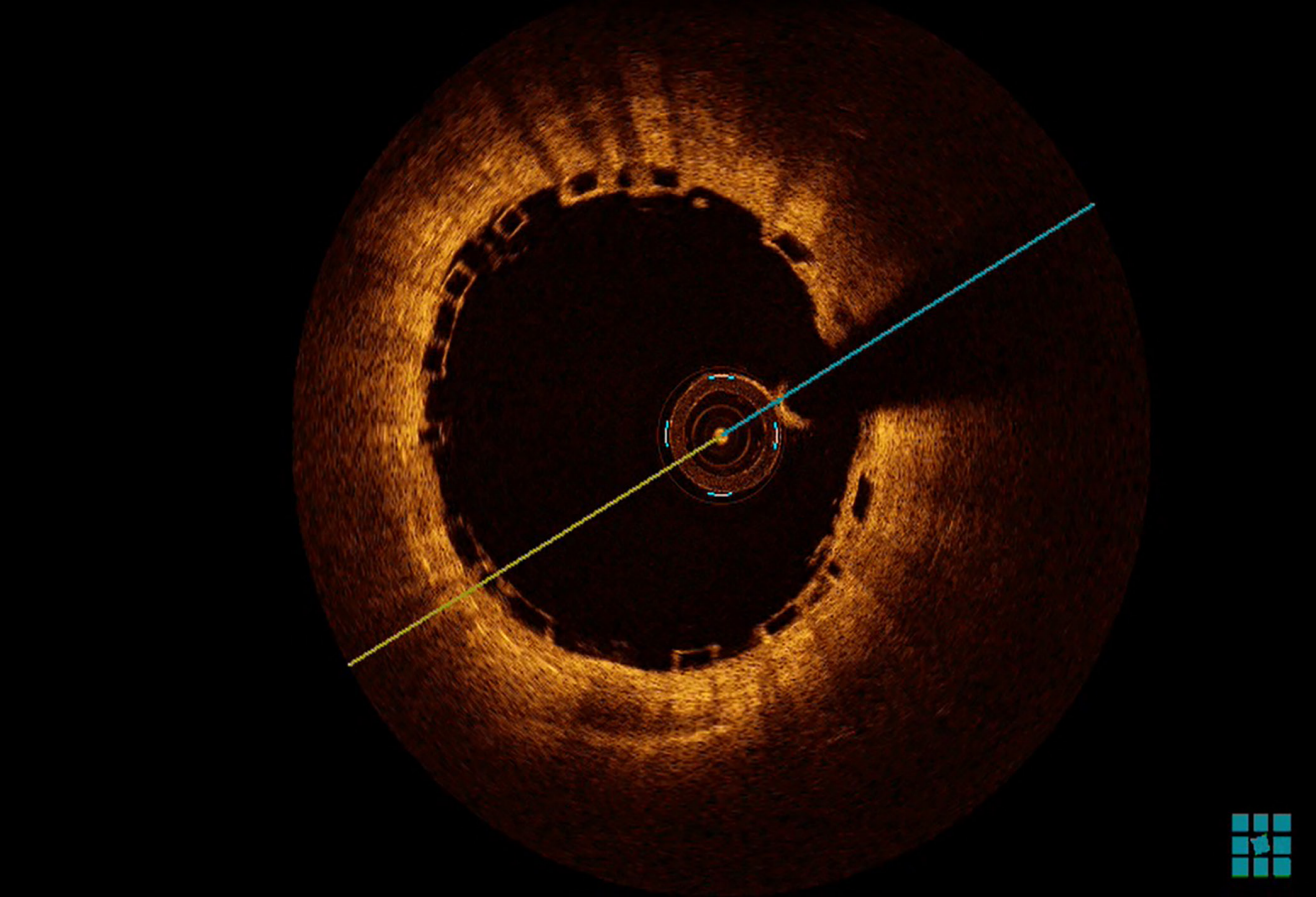
Optical coherence tomography run in left anterior descending artery during index bioresorbable scaffold implantation demonstrating the Check Box appearance of struts.

The coronary angiograms revealed only minimal plaques in RCA & LAD (Figure 1A and 2A). The BRS were identified on cineangiogram by their radio‐opaque markers at both ends (Figures 1B and 2B). OCT run of LAD artery revealed “ghost remnants” of BRS struts in LAD with a minimal stent area (MSA) of 7.0 mm^2^ (Figure 3). Meanwhile, the RCA BRS had completely healed with minimal “ghost” struts with an MSA of 5.5 mm^2^ (Figure 4). The ghost remnants of BRS resembled the original “Check box” appearance on OCT at implantation (Figure 5). The patient was advised medical follow up including DAPT. Fortunately, the patient remained angina free on follow up.

## 3 DISCUSSION

Drug eluting stents have revolutionized the management of coronary artery disease & acute coronary syndromes. Despite multiple advances, the problems of in‐stent restenosis & late stent thrombosis continue to plague metallic DES.^1^ BRS represents the next generation of stent technology with a multitude of benefits. By complete resolution of stent struts, it alleviates the risk of late stent thrombosis as well as curtails the need for prolonged DAPT.^2^ In addition, it restores vasomotion of vessel, preserves the opportunity for late positive remodeling and re‐intervention if needed.

ABSORB GT‐1 was the prototype Everolimus eluting BRS (Abbott Vascular, Santa Clara, CA) with maximum clinical data & an approval from the FDA for clinical use.^3^ After initial success of ABSORB I & II studies, the multi‐center ABSORB III trial failed to show superiority over metallic DES. After that multiple reports of Stent thrombosis appeared in literature and many causative factors were identified.^4^ Early cases of device thrombosis were ascribed to under‐expansion, undersizing, or geographical miss whereas scaffold discontinuity, malapposition, device recoil, neo‐atherosclerosis were prime contributors to late device dysfunction. Ultimately, ABSORB BRS was withdrawn from the market owing to low sales.

There were multiple shortcomings with the ABSORB BRS with three pivotal adversaries being high strut thickness (150 μm PLLA backbone with an additional 6‐7 μm coating of PDLLA), weak mechanical properties and prolonged resorption time (~ 3 years).^5^ Initially, it was claimed that the entire stent dissolutes by second year.^6^ From third year onwards, there is a gradual invasion of connective tissue from adjacent areas to fill the void left by resorbed polymer struts.^7^ The arrival of proteoglycan matrix heralds the final step in integration of stent into vessel wall. In OCT, the newly deployed stent gives a check box appearance on the luminal endothelium whereas unresorbed struts appear as “Ghost struts” or empty voids within vessel wall. Ideally, a completely dissolved BRS should not be discernible on OCT but in real world the struts were still visible at 2 years on OCT though they were nearly covered with endothelium.^8^

Apart from the device itself, patient‐ and operator‐related factors definitely play a role in long‐term outcomes. The use of a “P‐S‐P” strategy (Pre‐dilatation, correct scaffold Sizing, and Post‐dilatation) coupled with intra‐coronary imaging for scaffold deployment has been proposed to improve outcomes after BRS.^9^ However, in our case the operator(s) for both BRS were same, were implanted at same time and in the same patient. Hence, the differential resorption can only be attributed to the device only. The unresorbed struts pose a high thrombogenic risk and need for prolonged Dual anti‐platelet remains. Indeed, BRS implantation is now included as a high‐risk feature for stent thrombosis in guidelines necessitating prolonged DAPT.^10^, ^11^ Hence, one of the primal advantages of the BRS i.e., shorter DAPT has been antagonized by the delayed and erratic resorption of struts which has increased the thrombotic risk.

**FIGURE 6:**
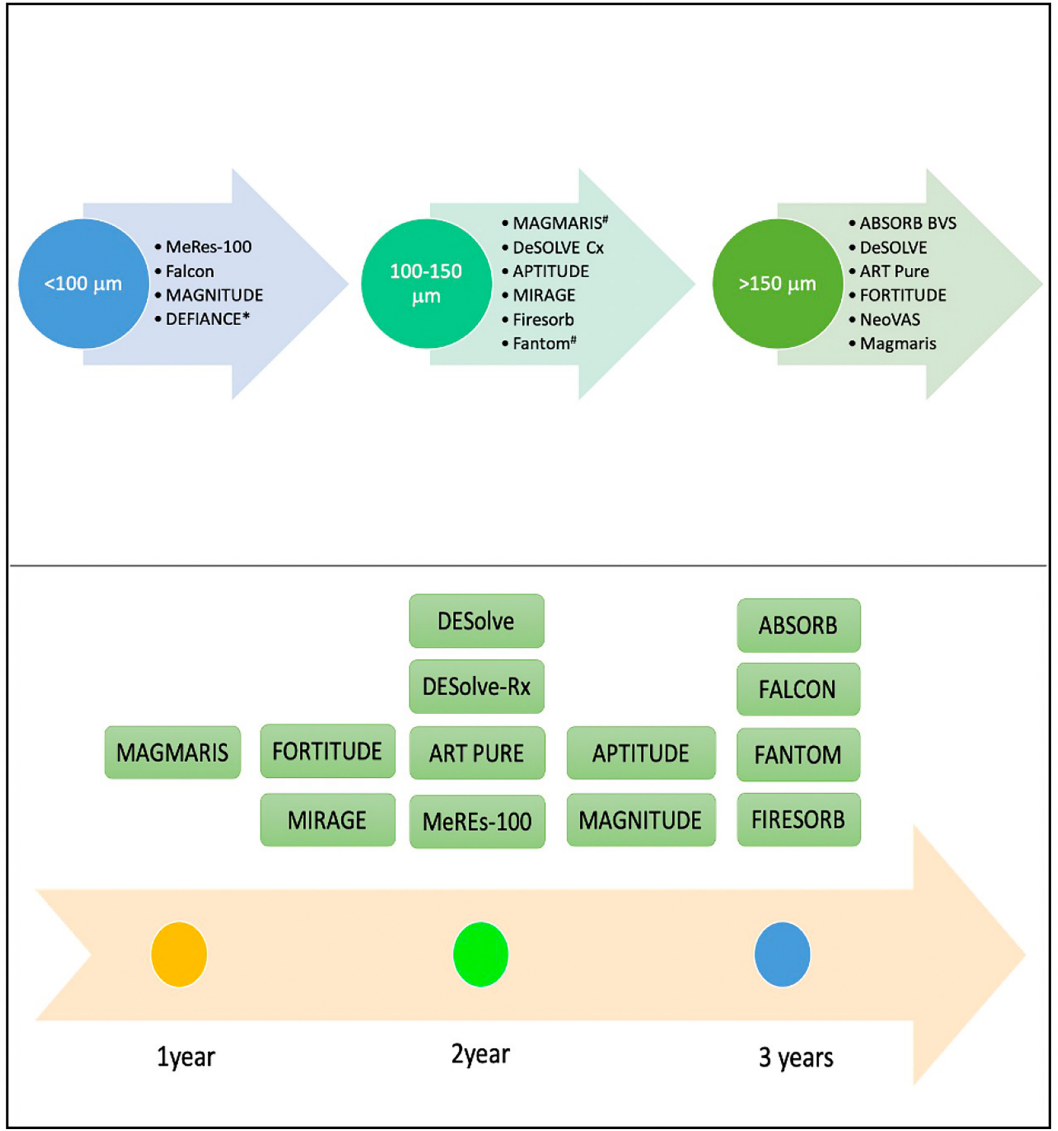
Comparison of two key characteristics of major bioresorbable scaffold in clinical development, or use—Strut thickness (Upper panel), and Strut resorption time (Lower panel). MeRes100 is approved in India for clinical use while DESolve, Magmaris, ART Pure, FORTITUDE, and Fantom are CE marked. All BRS elute drug except ART Pure which is drug free. Adapted from Ref. No.5,13,14. [*Defiance is the thinnest BRS available till date (85 m); ^#^Most BRS utilize the poly‐L‐ lactate platform (PLLA) or Poly D,L‐ lactate (PDLLA) except Magmaris (magnesium alloy based) & Fantom (Deamino‐ tyrosine polycarbonate)].

However, this is not a “class effect” and lesson learnt form failure of ABSORB are helping in refining more than 30 BRS in development from >20 different companies.^5^ Indeed, many new generation BRS do have strut thickness < 100 μm (MeRes100, Falcon, MAGNITUDE, and DEFIANCE (Figure 6)). DEFIANCE BRS (Amaranth Medical Inc., USA) is the thinnest at 85 μm. The usage of thinner struts can lead to higher penetration of the struts in wall and consequently lesser protrusion in lumen. This in turn has potential to promote better endothelisation and encapsulation of dissoluting struts by surrounding tissue attenuating late discontinuity. The APTITUDE BRS (115 μm; Amaranth Medical Inc., USA) has already shown better healing properties than ABSORB BRS in porcine arteries.^12^ The RENACENT II study (*n* = 60) established the safety and efficacy of APTITUDE BRS in humans with a high clinical (98%) and procedural success (100%).^13^ The angiographic late lumen loss was acceptable at 0.3 mm at 24 months. The RENACENT III is evaluated MANGITUDE BRS (98mcm, Amaranth Medical Inc., USA) in patients with CAD with myocardial ischemia (NCT02900937). The Interim 9‐month data (*n* = 70) presented at TCT 2018 demonstrated 97% device and procedural success with an acceptable late lumen loss of 0.28 mm.^14^ The 2‐year data was recently published and demonstrated the safety and efficacy of the device at 2 years.^15^ There was no scaffold thrombosis, and 97% struts were endothelialised. There was also a luminal gain of 1.6 ± 0.3 mm following BRS implantation. So, despite the minimal strut thickness its performance was similar to its thicker congeners (AMPLITUDE & FORTITUDE; Figure 6).

Newer BRS are evaluating platforms other than the classic Poly‐L lactide (PLLA) or Poly‐ D,L‐ lactide (PDLLA) utilized in ABSORB. The Magmaris BRS (Biotronik Inc., Germany) is an absorbable magnesium alloy‐based scaffold while the Fantom BRS (REVA medical, USA) uses a desaminotyrosine polycarbide base.^5^ Both these platforms enhance the radial strength of the BRS and make them less amenable to fracture during post dilatation.^3^ Additionally, the incorporation of iodinated tyrosine analogues in scaffold of Fantom BRS makes it more radio‐opaque throughout the resolution process. Thus, it offers dual benefits over its congeners facilitating precise deployment under fluoroscopy and obviating the dire necessity of imaging modalities like intravascular ultrasound and OCT. The FANTOM II demonstrated (*n* = 240) safety and efficacy of the device, high procedural success (97%) and low scaffold thrombosis (0.4%) at 12 months. The late lumen loss was acceptable 0.33 mm at 9 months.^16^

Other manufacturers (Amaranth Medical Inc., USA) have altered the properties of PLLA by heating and extrusion called as “Post processing” lead to formation of ultrahigh molecular weight PLLA polymers.^5^ Such ultrahigh molecular weight polymers lead to superior tensile strength (10 times higher elongation at break) and refractoriness to fracture during expansion (like post dilatation) compared to conventional PLLA and similar to metallic stents.^15^

Many newer BRS have resorption times less than that of ABSORB (around 3 years). This has advantages with curtailing the duration of dual antiplatelet with shorter resorption times and early restoration of vasomotion. Magmaris BRS has the shortest elution time of 9–12 months and showed high rates of vasomotion (80%) at 6 months in BIOSOLVE II study.^17^ The large BIOSOLVE IV trial (*n* = 1024) has established safety and efficacy of Magmaris BRS in broad patient population with low scaffold thrombosis (0.5%) at 1 year.^18^ However, such faster resorption time could be counterproductive with an increased risk of vessel recoil and restenosis specially magnesium based BRS.^19^ The resorption patten is also different with metallic & nonmetallic BRS. While the polymer based BRS degrade by a nonenzymatic process and both surface/ interiors resorb simultaneously.^5^ In contrast, the metallic BRS, start eroding from surface inwards but the exact clinical implications are yet to be observed on ground in head‐to‐head studies.

All BRS use drug elution except ART Pure which is devoid of drugs. Currently, MeRes100 is approved for clinical use in India while five (ABSORB, DESolve, ART Pure, Fantom, and Magmaris) are CE approved for use under clinical trials.^20^, ^21^ The European association of PCI (EAPCI) in conjunction with European Society of Cardiology (ESC) has also laid down a structured framework for evaluation of newer BRS in future.^21^ They recommend a follow up period in trials well beyond the expected resorption time of the BRS. This will aid in demonstration of long‐term safety of device and allow assessment for vessel patency, neointimal growth, and residual inflammation after resorption of device. They also advocate a prolonged DAPT of 36 months for patients in whom ABSORB BRS is already implanted. As an additional safety measure, it would be prudent to do an intracoronary imaging (if feasible) to demonstrate absence of “Ghost Struts” and safely discontinue dual antiplatelet therapy. The major learnings from this case include mandatory application of intracoronary imaging and attention to proper technique (“P‐S‐P”) both for device optimization while implanting a BRS.

## 4 CONCLUSION

The equipoise between strut thickness, resorption time, and ideal scaffold platform for resorption is yet to be achieved. After the failure of ABSORB, many other devices are trying to build upon the lessons learnt from its debacle. Many BRS platforms have shown encouraging results, but the war is far from over. We will have to wait for the unequivocal results from a large and sufficiently powered RCT before BRS re‐enters the catheterisation laboratory as a workhorse device.

## AUTHOR CONTRIBUTIONS

**Akshyaya Pradhan:** Visualization. **Shubhajeet Roy:** Resources. **Monika Bhandari:** Data curation. **Pravesh Vishwakarma:** Investigation. **Marco Alfonso Perrone:** Conceptualization; writing – original draft. **Rishi Sethi:** Data curation. **Md. Al Hasibuzzaman:** Conceptualization; methodology; project administration; writing – original draft.

## FUNDING INFORMATION

None.

## CONFLICT OF INTEREST STATEMENT

None.

## CONSENT

Written informed consent was obtained from the patient to publish this report in accordance with the journal's patient consent policy.

## Data Availability

The data that support the findings of this study are available from the corresponding author upon reasonable request.
